# Supplementary figures and images for: Connectomics of the Octopus vulgaris vertical lobe provides insight into conserved and novel principles of a memory acquisition network
Source: eLife. 2023 Jul 6;12:e84257. doi: 10.7554/eLife.84257 (PMC10325715; doi:10.7554/eLife.84257)

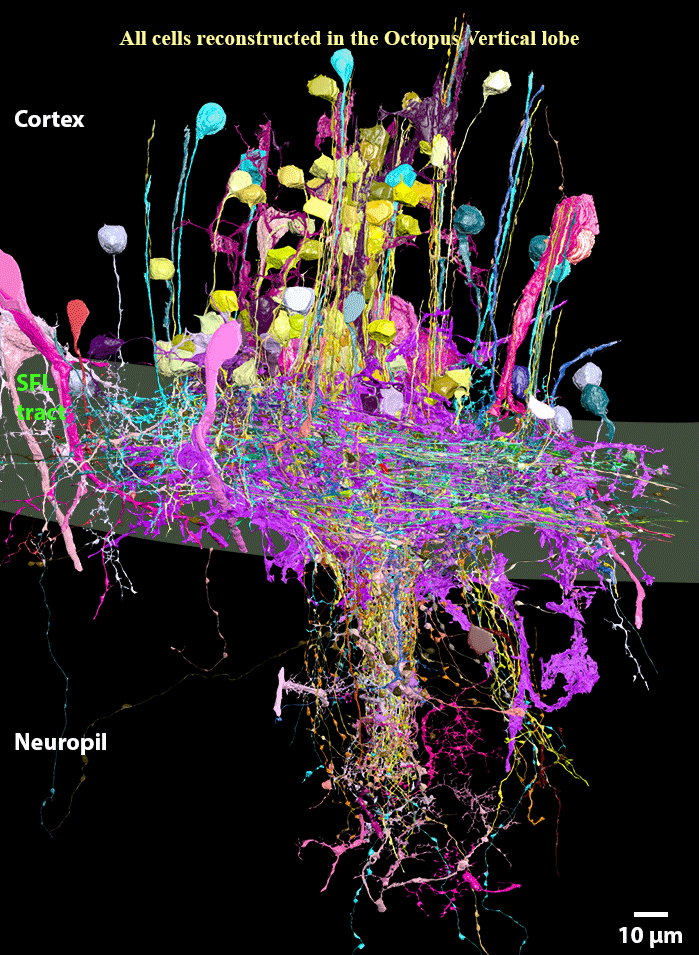

Supplement: Supplementary file 2 [file elife-84257-fig1-video2.gif]
